# Pressure–Induced Cell Wall Instability and Growth Oscillations in Pollen Tubes

**DOI:** 10.1371/journal.pone.0075803

**Published:** 2013-11-19

**Authors:** Mariusz Pietruszka

**Affiliations:** Faculty of Biology and Environment Protection, University of Silesia, Katowice, Poland; University of Zurich, Switzerland

## Abstract

In the seed plants, the pollen tube is a cellular extension that serves as a conduit through which male gametes are transported to complete fertilization of the egg cell. It consists of a single elongated cell which exhibits characteristic oscillations in growth rate until it finally bursts, completing its function. The mechanism behind the periodic character of the growth has not been fully understood. In this paper we show that the mechanism of pressure – induced *symmetry frustration* occurring in the wall at the transition-perimeter between the cylindrical and approximately hemispherical parts of the growing pollen tube, together with the addition of cell wall material, is sufficient to release and sustain mechanical self-oscillations and cell extension. At the transition zone, where symmetry frustration occurs and one cannot distinguish either of the involved symmetries, a kind of ‘superposition state’ appears where either single or both symmetry(ies) can be realized by the system. We anticipate that testifiable predictions made by the model (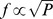) may deliver, after calibration, a new tool to estimate turgor pressure 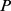 from oscillation frequency 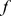 of the periodically growing cell. Since the mechanical principles apply to all turgor regulated walled cells including those of plant, fungal and bacterial origin, the relevance of this work is not limited to the case of the pollen tube.

## Introduction

### General outline

The pollen tube has become a widely used cellular model system. In addition to being one of the fastest growing plant cells, it features periodic oscillations of the growth rate that have attracted numerous attempts to model the process. While recent models have increasingly incorporated biological features such as ion transport and intracellular trafficking, a simple feature with potentially significant impact has been overlooked in past approaches, namely: geometry. We modeled the strain rates in the cell wall caused by turgor pressure as a function of the different symmetries present in the pollen tube and found that a crucial area on the cellular surface of the pollen tube can be characterized by what we have termed *symmetry frustration*. (The term frustration, in the context of magnetic systems, has been introduced by Gerard Toulouse [1]. Early work includes a study of the Ising model on a triangular lattice with nearest-neighbor spins coupled antiferromagnetically [2]). This area represents the transition zone between the hemisphere-shaped apex and the cylindrical shank. From a biological point of view this zone is crucial since numerous molecular landmarks of polar growth are present on one side of this zone and are absent from the other.

The model predicts that the transition zone undergoes local peaks in strain rate, revealing intriguing possibilities for research on the polarity of the growth process. Furthermore, we propose that changes between different symmetry regimes might be the mechanical underpinning of periodic changes in growth rate and shape observed during oscillatory growth. We believe that our model makes an important contribution to the field of plant cytomechanics in general and pollen tube growth in particular.

### Preliminaries

Pollen tubes are rapidly growing plant cells whose morphogenesis is determined, at least in part, by spatial gradients in the biochemical composition of the cell wall. Pollen tube growth is a critical process in the life cycle of higher plants [3]. It has garnered a lot of attention and is at the center of considerable controversy [4]. The pollen tube is the carrier of the male gametes in flowering plants. The controversy involves the modes of extension leading to periodicity in growth and growth rate [3]. While some authors claim that hydrodynamics is the central integrator of pollen tube growth [5], [6], [7] leading to growth oscillations, others relate the periodicity in growth dynamics to the changes in the wall material properties [3], [8], [9].

**Figure 1:**
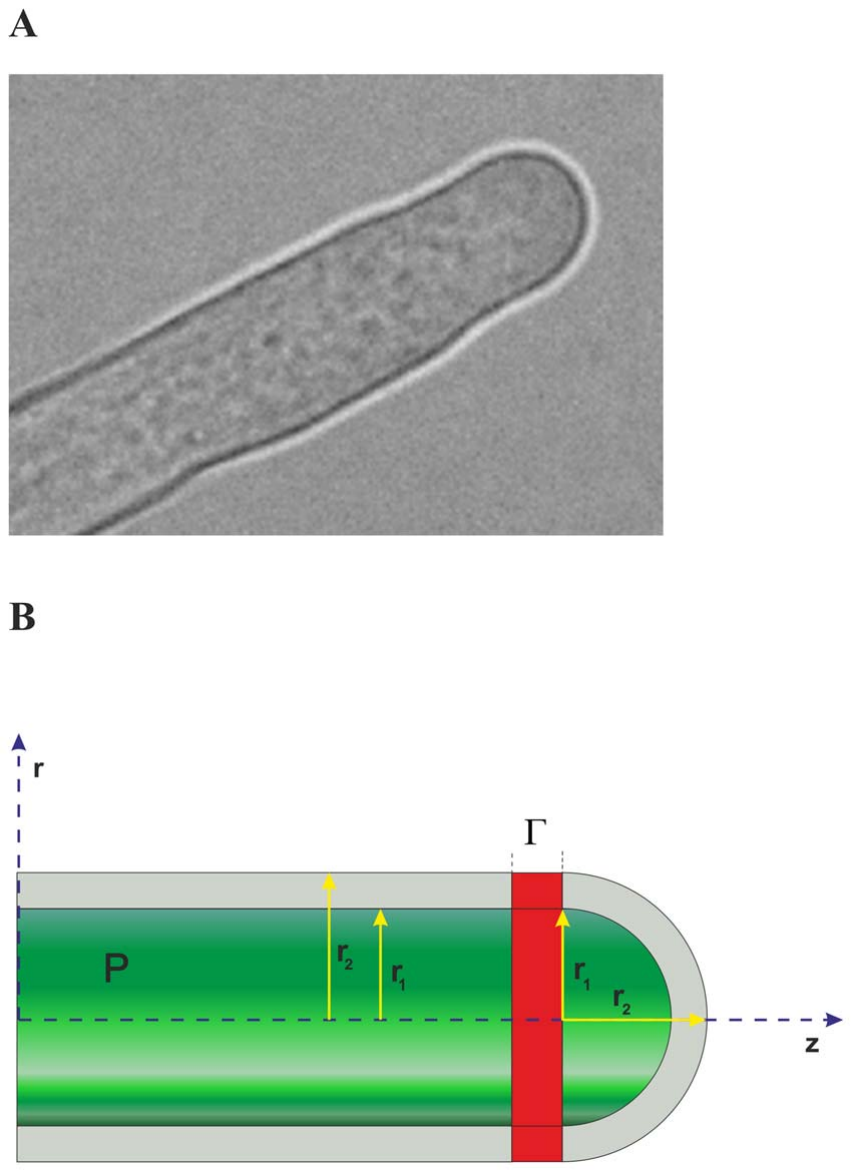
*Nicotiana tabacum* pollen tube apical region. (A) Microscopic view (B) Schematic view: radii of curvature 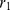 and 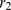, turgor pressure 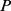 and the investigated partition into two distinct symmetry regions are indicated in the chart. Note, the ‘transition zone’ 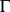 can also exist on the spherical part, or both.

**Figure 2:**
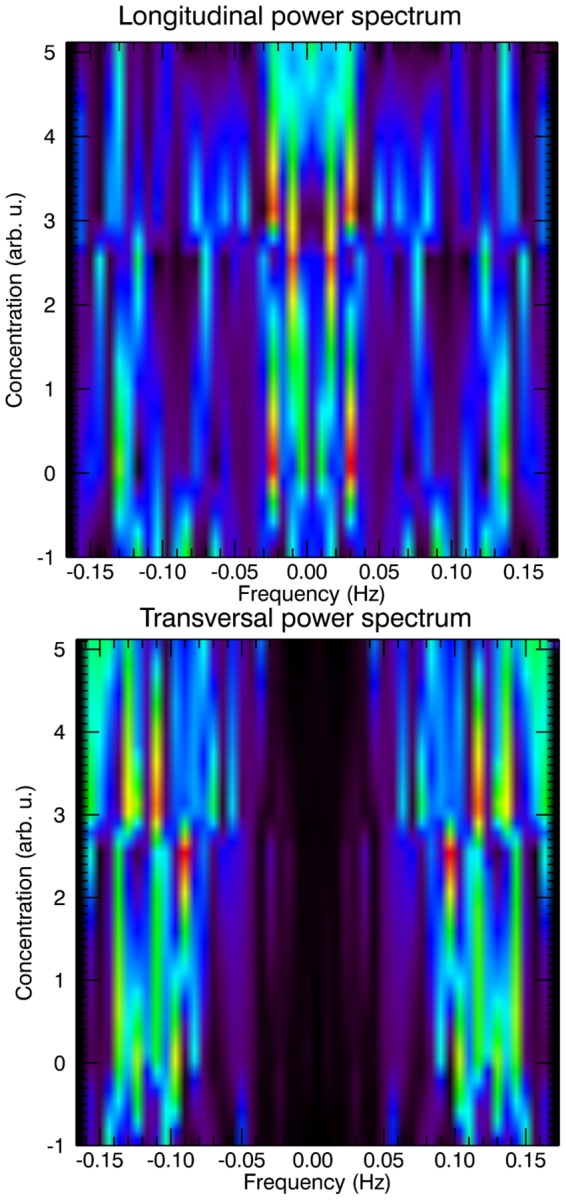
Density plots of the longitudinal and transversal power spectrum of an individual *Nicotiana tabacum* pollen tube. Graphs obtained from the raw experimental data, bias subtracted [14] by Fourier analysis, calculated by the power of the Nyquist criterion and Nyquist rate, for different osmotic environments (−1 corresponding to the hypotonic case, 0 – isotonic case, and 2.5, 3 and 5 corresponding to 25, 30 and 50 mM NaCl in hypertonic conditions, respectively). A broad, narrowing valley at the centre of the lower plot is clearly visible – low frequencies seen for longitudinal modes are shifted outwards. Red colour indicate high intensity peaks. Interpolated by DAVE, developed at NIST [59].

Pollen tubes are tip growing cells, which means that cell volume increase is confined to the tip of the cell. They display extremely rapid growth that can be also reproduced *in vitro*. All activities associated with wall growth - including delivery of new cell wall material and cell wall deformation - occur exclusively at the hemispherical tip of the cell [10] (Fig. 1A). Deformation of pre-existing wall material is driven by the turgor pressure, a hydrodynamic pressure inside the cell. Growing pollen tubes characteristically display characteristic oscillations in growth and growth rate [11], [12], [13]. These growth oscillations depend on many phenomena, among which are the underlying ion and mass fluxes, wall mechanical properties, system symmetries and turgor pressure. In isotonic conditions [5] the average growth cycle period 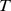 is about 50 s, whereas in hypertonic or hypotonic conditions it shifts to about 100 s and 25 s, respectively. The latter produce oscillations with typical frequencies (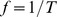): hypertonic – 0.01 Hz, isotonic – 0.02 Hz and hypotonic – 0.04 Hz. The longitudinal and transverse oscillation power spectrum of an individual *Nicotiana tabacum* pollen tube [14] is visualized in Fig. 2, and can not solely be described by a double exponent model (e.g. Fig. 8A in [15]), which, although suitable for normal cell enlargement, but insufficient for periodical growth.

The cell wall is one of the key structural players regulating plant cell growth since plant cell expansion depends on an interplay between intracellular pressures and the controlled yielding of the wall [16]. Cell wall polymers are amorphous polymers existing above their ‘glass’ transition temperature, so that considerable segmental motion is possible. At ambient temperatures, the cell wall is thus relatively soft (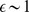 MPa) and is easily deformed. Cells can grow to some extent simply by stretching their walls as they take up water. However, continued cell expansion involves synthesis of new wall material. Synthesis of cellulose at the plasma membrane and of pectin and hemicelluloses components with Golgi apparatus deposits successive layers on the inside of the existing cell wall. A mechanical prerequisite for the unidirectional growth of pollen tubes for the (scalar) hydrostatic pressure is a softer cell wall at the tip of the cell, and a more rigid wall at the basal part [17], [18]. This gradient of mechanical properties is generated by the absence or scarcity of callose and cellulose at the tip [19] as well as by the relatively high degree of esterification of the pectin polymers in this region. The gradient in cell wall composition from apical esterified to distal de-esterified is reported to be correlated with an increase in the degree of cell wall rigidity and a decrease of visco-elasticity [20]. Microindentation studies confirm (Fig. 2 in [3], [21]) that the pollen tube tip is less rigid and that the distal stiffness may be opposed to apical softness. Needless to say, in order to sustain growth, a balance between the mechanical deformation of the viscoelastic cell wall and the addition of new cell wall material must be achieved [22].

Turgor pressure is the pressure of the cell sap against the wall in plant cells. This is a force exerted outwardly on the plant cell wall by the water and solutes contained therein, giving the cell a hydrostatic rigidity. Excess turgor pressure or local cell wall weakening can result in the bursting of a cell. Both, turgor pressure and the wall properties are decisive for the mechanical behaviour and dynamics of the developing plant cell [23]. In fact, the physical properties of the wall and the turgor pressure have pivotal functions since they represent the “downstream parameters” of all cellular signaling events [24]. For our future reference we note that the turgor pressure in lily pollen tubes ranges from 0.1–0.4 MPa in lily [25], [3].

Pollen tube geometry can be described by two different symmetries - a hemisphere shaped apex, and a cylindrical shank connected by a transition zone between the two. In this paper we describe a mechanism of ‘symmetry frustration’ occurring in this transition zone between the two involved symmetries as a possible mechanism responsible for growth rate oscillations. In simple terms by symmetry frustration we mean that a small ring of cell wall (hereafter referred to as interface 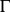, Fig. 1B) is unable to ‘decide’ if it should behave as an elastic cylinder or an elastic sphere. Following this hypothesis, oscillations may arise because this mesoscopic ring at the equator behaves as if it ‘jumps’ periodically between the two mechanical states of different strain energy capacity.

The application of growth tensors to developing plant organs has been known for a long time [26]. Such mathematical descriptions has been used to model apical meristems where the proliferating cells produce tissue stresses, which in turn influence the structure of the developing organ and therefore determine the principal directions of growth [27]. As one might expect, various stresses occur along the length of any tip-growing cell because of the varying properties of cell wall from one region to another when exposed to the turgor pressure [28]. The distribution of the wall stresses as well as deformation of the particular wall layers can in principle be calculated by solving equilibrium equations of elasticity theory. The equilibrium equation may be derived both for materials deformed elastically (deformation vanishes when force equals zero) or non-elastically (plastic deformation survives, even when the acting force is removed). In this article we concentrate on elastic properties, because the oscillatory growth of pollen tubes is our main concern [7], [9], [8]. Plastic properties are inherent in the proposed model *via* the derived (anharmonic) ‘frustration potential’, and the assumed cell wall building processes located in the sub-apical, annular region, presumably at (above) the 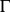 – interface [29], [30]. As an aside, note that whereas the hydrodynamic model as it is used by Zonia [7] proposes gradual increase in turgor until a threshold when rupture of individual links between cell wall polymers occurs, Winship and coworkers [3] state that turgor is essentially stable, but an exocytosis–induced relaxation of the wall causes expansion. They postuale that variations in cell wall mechanical properties cause the oscillations and that variations in turgor (if there are any) are a passive consequence due to cell wall relaxation.

Several possible mechanisms have been proposed to account for the behaviour of growing pollen tubes in quantitative terms [31], [13], [32]. A model for calcium dependent oscillatory growth in pollen tubes has been put forward by Kroeger et al. [33]. More recently, Fayant et al. used a finite element technique [18] to establish a biomechanical model of polar growth in walled cells. Other models have included a chemically mediated mechanism of mechanical expansion of the pollen tube cell wall in which deposition causes a turnover of cell wall cross-links, thereby facilitating mechanical deformation [34]. The possible role of wall ageing in the self-regulation of tip-growth was considered in [35]; while a model based on plasma membrane flow and cyclosis regulation in growing pollen tubes was discussed in [36]. Campas and Mahadevan (2009) treat the irreversible expansion of the cell wall during growth as an example of the extension of an inhomogeneous viscous fluid shell under the action of turgor pressure, fed by a material source in the neighborhood of the growing tip [37]. Lastly, a realistic osmotic model of the growing pollen tube has been proposed recently by Hill et al. [38]. However, none of the models produced oscillations on mechanical basis. Indeed, the model presented in [36] actually does not describe the cell wall, and hence, can not predict oscillations. The authors assume that the growth oscillations are given to understand the phase angle differences between growth velocity and the regulating mechanisms. But yet, by definition, oscillation is the repetitive variation, typically in time, of some measure *about* a central value (often a point of equilibrium), and pollen tubes do not exhibit such form of oscillations. In fact, what we observe in pollen tubes is a periodical elongation (without shrinking phase), and can be a result of transitions between two or more different states, which is another definition of oscillation, we adopt in this paper.

Our approach does not invalidate previous studies. We explore the relationship between turgor pressure and nontrivial cell geometry in terms of mutually exclusive instability models [39] based on cylindrical and spherical geometries, both of which are present in rapidly extending pollen tubes. We base our physical model on the parametrized description of a tip-growing cell that allows the manipulation of cell size, cell geometries, cell wall thickness, and local mechanical properties. However, the mechanical load (contrary to op. cit.) is applied in the form of constant hydrostatic pressure.

An important feature of pollen tubes elongation is the observation that the growth rate oscillates and that additionally, many of the underlying processes also oscillate with the same fundamental period, but usually with different phase (e.g. Fig. 1c in [7]). The possible roles of these oscillating ion gradients and fluxes in the control of pollen tube growth [40] is beyond the scope of this paper and will not be discussed here. We share the view expressed in [34], [41] that the wall extension is primarily a biophysical (mechanical) process, and although ultimately dependent on enzymatic activity, under conditions where the enzymatic background can be subtracted out the biophysical process still proceedes normally. The origin of the oscillation is still unclear, though all hypotheses agree in that, the cell wall mechanics are essential to the oscillation [36].

Any new model should deliver testable and quantitative predictions that can be validated by experimental data. In case of pollen tubes, it is necessary to present predictions that go beyond stress values which are inherently difficult to measure. The presented model satisfies this requirement by offering, among other things, an experimentally testifiable power law (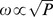) which relates the turgor pressure 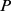 and the oscillation angular frequency 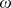.

## Results and Discussion

In order to construct a stress-mechanical model of observed pollen tube growth oscillations we begin with an analysis of the mechanical properties of a tip growing cell. In particular we focus on the circular perimeter located at the junction of hemispherical dome and the cylindrical shank. This is based on the observation that cell wall assembly by exocytosis occurs mainly in an annular region around the apical pole of the cell [42], [6] and that the concomittant turgor driven deformation of the cell wall causes characteristic strain in the hemisphere shaped apex of the cell [18], [34]. The dynamic properties of such a complex growing system should be self-consistent (meaning that the turgor pressure and the wall mechanical properties are conjugate magnitudes that usually form coupled equations, which have to be solved iteratively). Nevertheless, in the first approximation the following heuristic solution can be proposed.

Assuming an intrinsic and constant turgor pressure 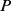 [38], and a much lower external pressure, presumably atmospheric pressure producing an effective pressure within the wall material 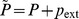, the equilibrium equation for the displacement vector 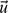, which is the shortest distance from the initial to the final position of a moving point, takes the form [43]

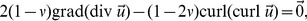
(1)where 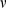 is the Poisson coefficient. For our purposes Young's modulus 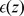 and Poisson coefficient 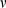 are assumed to be picewise constant functions, so they remain constant on the interface 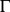 (Fig. 1a); “grad”, “div” and “curl” are differential operators, here acting on the displacement vector 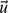.

Note, that by acting divergence operator on both sides of Eq. (1) we receive 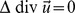, i.e. 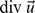 denoting the volume change due to displacement field is a harmonic function, i.e. satisfying Laplace's equation.

Eq. (1) may be solved analytically providing that the problem exhibits a high degree of symmetry. In particular it can be solved exactly for both spherical and cylindrical symmetries given the fact that both symmetries are necessarily present (Fig. 1B). It follows that the symmetries of both subdomains must be used in any description of pollen tube shape and dynamical properties.

By assuming cylindrical symmetry (for the displacement vector field 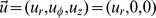, which is obtained under the assumption that the total length of the cylinder part remains constant (the axial elongation of the more rigid shank upon application of a constant internal pressure 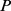 we assume as negligible), and hence (in first approximation) we accept 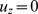 in 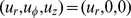 instead of 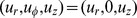, which would lead to unavoidable numerical solution of Eq. (1)), and representing field operators (grad, div and curl) in cylindrical (polar) coordinates, Eq. (1) can be reduced to a much simpler form:
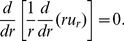
(2)This differential equation can be solved for the displacement field 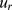 to yield the displacement for the cylindrical symmetry
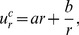
(3)where 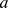 and 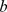 are constants to be determined from the boundary conditions [43]. For displacement in the spherical tip the introduction of spherical coordinates with the origin in the center of a sphere allows us to define the displacement field 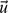 as a function of the radius 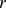: 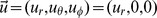. Therefore 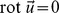 and Eq. (1) reads: 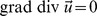. Hence for displacement spherical symmetry yields
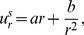
(4)The upper index in Eqs (3) and (4) has been substituted to differentiate solutions for cylindrical (c) and spherical (s) geometries. With these assumptions a realistic cell geometry for an elongating pollen tube can be described in terms of a cylinder of radius 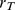 capped by a prolate half-spheroid with short radius 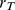 and a long radius 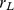 ([18]; Fig. 1). Our working model for pollen tube growth thus consists of a thin-walled hollow cylinder ending by a hemispherical shell immersed in an external pool of pressure 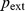 and filled with a cell sap with turgor pressure 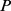 (we equate both radii 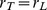, for simplicity). The inner radius of the cylinder and the sphere is 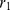, while the outer radius is 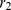 (Fig. 1B). Another simplifying assumption is that we deal with weak (elastic) interactions at the interface 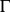, because of ongoing wall building processes resulting in the fact that the deflection field may differ slightly on both sides of the interface. From the mechanical point of view the two domains may be treated as being weakly coupled. This is consistent with the view that new deposition is associated with wall-loosening whereby load–bearing cross–links are broken, while simultaneously creating new load–free cross–links; thereby effecting a fail-safe scenario for mechanical expansion [34].

It has been proposed [44], [33] that mechanical properties of the cell wall at the growing tip must be different from those in the shank, and suggested that therefore an anisotropy in the cell wall elasticity is required to account for the transition between spherical and tubular shape at the tip of the cell. It has also been documented [45] that the rigidity of the tip of the pollen tube increases with increasing distance from the apex. Therefore, in a first approximation the elastic properties of our cylinder modeling the cell wall at the shank, and our hemisphere modeling the tip, are represented by two pairs of material constants: Young's modulus 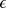 and Poisson coefficient 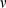. Further calculations may assume different, and perhaps more representative values for these coefficients for distal (thick and rigid) and apical (thin and elastic) walls of a pollen tube. Nonetheless, the proposed effect of geometrical frustration will be preserved even for slow gradient or other monotonic behaviour (along the long axis) of elastic parameters at the vicinity of the equator 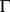. In other words, the effect of a continuous gradient of Young modulus and Poisson coefficient in a broad transition zone that engulf both the shank and the apical dome on the symmetry frustration and growth oscillations is of minor weight. Assumptions, about different values of mechanical constants at the apical dome and cylindrical shank, are fully justified (see e.g. Fig. 4 in [45]), where spatial distribution of the Young's modulus is presented). Because we consider relatively small elastic deformations, the stress and strain tensors are related by the Hooke's law of elasticity making the deformation reversible. For the radial part of the stress tensor 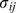 we have: 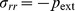 at 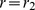 and 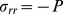 at 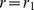. Since the off-diagonal elements vanish, we are left with the strain 
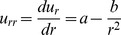
, 
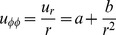
 and 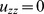, the radial 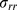 element of the stress tensor reads:
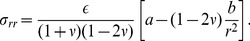
(5)By assuming boundary conditions as above, parameters 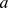 and 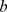 can be calculated [46]. They both depend on the material constants: Young's modulus 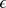 and Poisson coefficient 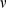, cylinder geometry (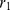, 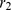 – radii) and on pressure values 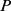 and 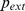. In fact, to receive smooth solutions on 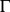 – interface equations for different geometries should be connected by transmission (gluing) conditions equating the forces and deflections on each side: 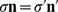 and 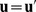, where 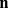 denotes the exterior normal to the boundary. In first approximation we let both subdomains be weakly coupled (visco – plastic phase) while cyclic wall building processes take place at 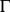, and strongly coupled mechanically (visco – elastic phase) when wall building processes expire. We consider only the radial part of the stress 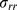, deflection 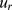 and strain 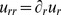 tensors on 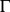, for simplicity. This Ansatz, however, does not qualitatively influence the results.

Quantitative calculations stemming from Eqs (3)–(5) originating from the gradient of mechanical properties at 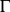 are presented in File S1 (see also Figures S1, S2, S3, S4, S5 in File S1: Fig. S1 – Displacement 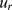 due to the effective turgor pressure 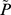 acting on the cell wall as a function of the radial distance 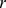 from the pollen tube long axis; Fig. S2 – Tensile stress 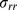 due to the effective turgor pressure 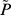 acting on the cell wall at the position where the cylinder (shank) joins the hemisphere (apex) as a function of the radial distance 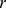 from the pollen tube long axis; Fig. S3 – Tensile stress difference 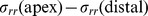 at the apex and the distal part; Fig. S4 – Tensile stress difference 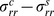 calculated at the boundary zone between the approximately hemispherical apical and the cylindrical distal part of the growing pollen tube (parametrisation by the turgor pressure); Fig. S5 – Tensile stress difference 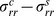 calculated at the boundary zone between the semispherical apical and the cylindrical distal part of the growing pollen tube (parametrisation by the wall thickness)).

**Figure 3:**
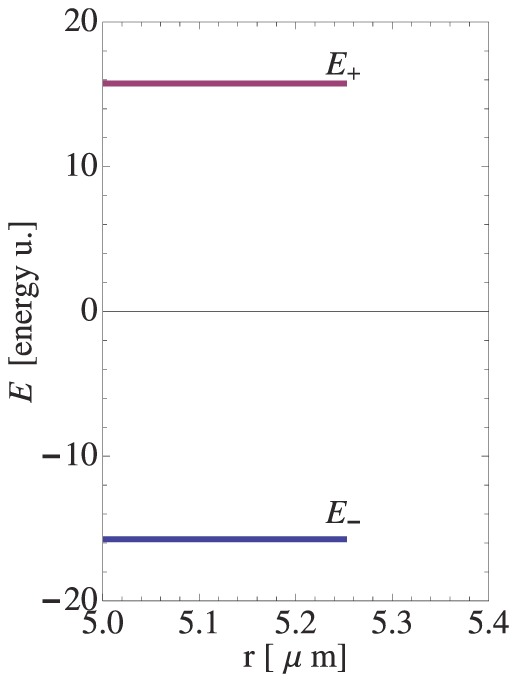
Frustration – induced energy splitting in a pollen tube apical region. Corresponding symmetry exchange takes place between the resonating residual energy levels 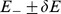 and 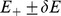 of different major symmetry. Calculation performed at the transition zone 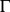 between the (hemi-spherical) apical and the (cylindrical) distal part of a growing pollen tube (see Eq. (7)), at a constant turgor pressure 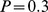 MPa. The inner and the outer wall radius in both subsystems read: 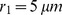, 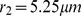 (wall thickness 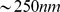), respectively. The dispersion of each energy level 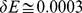. For bifurcation diagram and tensegrity force see arXiv:1211.1143.

**Figure 4:**
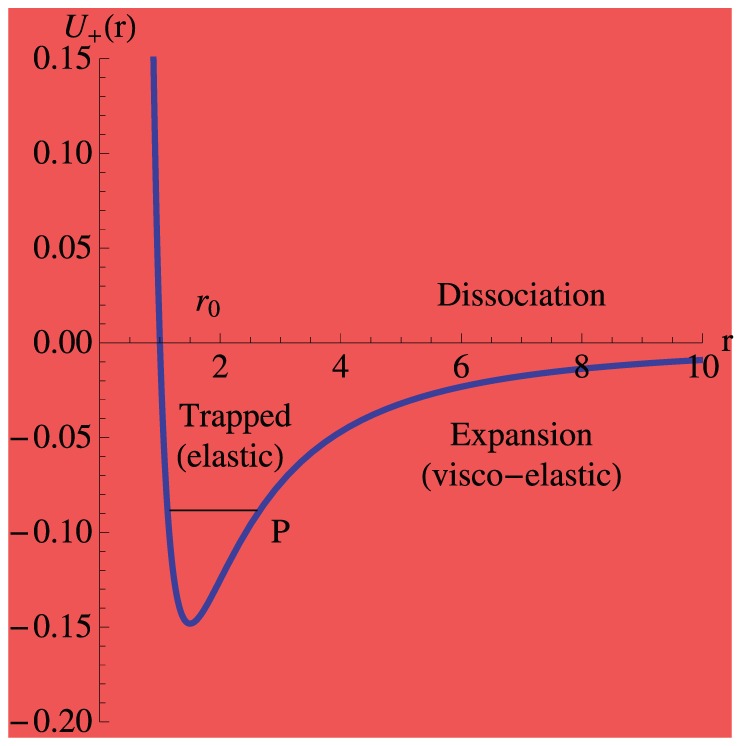
One of the two branches of the anharmonic (‘frustration’) potential 
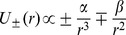
, as a function of wall constituing molecules separation 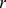. (Here: 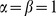; in general the coefficients 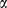 and 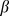 are linear in 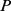, see Appendix S1). Oscillations take place between 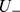 and 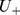 potential energy level (compare with [60], Fig. 2), by tunneling through symmetry change, yielding 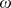, while the amplitude is determined by the actual pressure 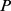 level, in accord with experiments ([4], [9]). The source of the potential: pressure – induced (residual) internal stress field in the wall. See also arXiv:1211.1143.

On the other hand, because curvature discontinuity and stress singularity both occur at the transition zone the expression for tensile stress difference due to the symmetry change 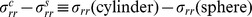 which becomes
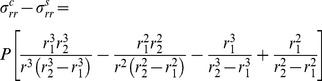
(6)and conversely from the opposite formula: 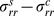, it is shown that calculations performed for 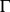 - interface, where both geometries (cylindrical and spherical) meet, a *symmetry frustration* – leading to oscillations of the radial part of the stress tensor – may take place. The term ‘frustration’ signifies the fact that none of the locally involved symmetries is distinguished. On the other hand, (by evaluating 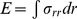) from Eq. (6) the calculated strain energy density reads:
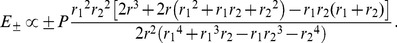
(7)The quasi–discrete energy levels 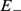 and 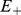 (possesing a small dispersion 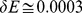 [energy u.]) presented in Fig. 3 are non–degenerate due to the constant turgor pressure 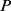, which leads to the observed splitting of energy levels. Still, since both levels originate from the symmetry change at 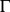, they can be account for the oscillations in the pollen tube growth functions. Thus, the *resonant frequency* of growth (growth rate) corresponds to the energy difference 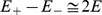 (since 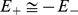), which in turn is directly proportional to the turgor pressure 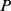. Consequently, we can equate the transition energy 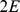 between the resonating levels (Fig. 3) with the pollen tube oscillation frequency observed in experiments. We note that Eq. (7) implies that if 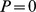 then the system exhibits no oscillations, which is exactly the case. (See also the plot of the potential energy 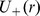 at 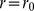 in Fig. 4).

**Figure 5:**
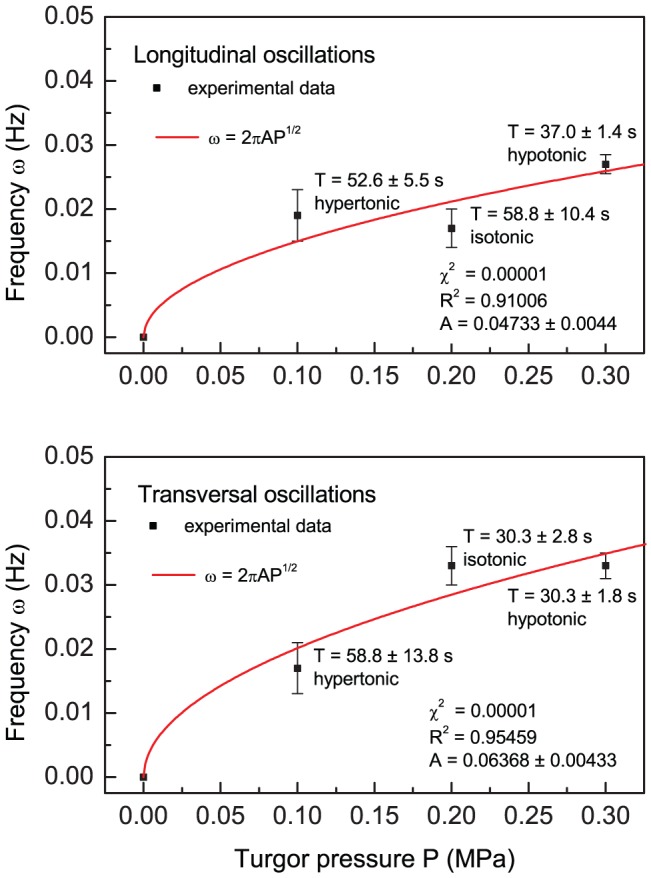
Least square fit of the experimental data (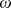) as a function of turgor pressure 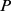. Data collected for hypertonic (25 mM NaCl), isotonic and hypotonic (hypo-osmotic stress induced by the addition of water to the gel cultures) treatment of *Nicotiana tabacum* pollen tube [14] are fitted to the square root function derived in this paper (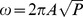; 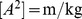). Stable turgor values (implemented equidistant measures) correspond to those ranging between 0.1 and 0.4 MPa, which has been also recorded using a turgor pressure probe [25]. The initial point 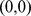 is an exact physical constrain, as the pollen tube will not oscillate (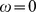) for the vanishing turgor pressure (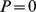).

Notwithstanding, we note that the considered effect is exclusively connected with geometrically induced stress in the wall, which may be linked with symmetry frustration, (see [18], Fig. 4F) and one can express it in measurable units [Pa m]. A spatially degenerate ground state will undergo a geometrical distortion (the alteration of the original shape) that removes that degeneracy, because the distortion lowers the overall energy of the whole complex. Indeed, in calculating the definite integral over the function expressed by Eq. (6): 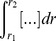 (with 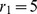 and 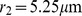) we obtain a strain energy density: 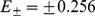 [MPa 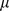
m]. Therefore, the difference of strain energy density between the two levels is about 0.5 MPa for micrometer length scales typical for the width of the pollen tube cell wall (about 250 nm). Such energy densities may lead to oscillations, which are observable not only in growth rates. From our model it can also be inferred that the apical geometry oscillates (due to deformation 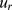 located initially at 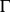, compare Figs. 3B and 4B in [47]), to produce a so called “pearled” morphology [34]. Evidence of such deformations at 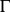 can be seen in Fig. 1c and 6a, c (ibid.) as crests smeared out on a distance 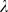, in agreement with our model. Such geometrical oscillations of the wave–length 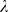 will be obtained when frustration occurs, and the cylindrical and spherical symmetries will be present on 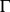 – contour interchangeably; compare Movie S1 and S2 in [47]. However, what we also note that there is no sign of deflection at a distance shorter than the tube radius 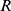. The latter observation further supports the main idea presented in this paper of specific role of the 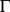 – interface in initiating oscillations. By assuming, after [34], the value of the linear velocity 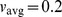 [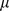
m/s] of the elongating cell and taking the average oscillation period 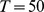 s from Fig. 5 we can confirm the observed wavelength of about 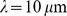, which is a doubled value of the radius 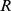, as expected assuming the correctness of our approach. In this picture, the wavelength 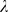 includes the times necessary for local deflection, wall stress/stress relaxation, and recovery through wall building processes for every period 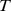.

Transitions between the different states of symmetry are shown in Fig. 3. The system is pumped with energy sufficient to jump over the energy gap between the (hemi-)spherical and cylindrical geometries. A positive feedback mechanism is necessary to drive the oscillations and to prevent damping due to viscosity. The necessary additional energy is absorbed (presumably generated by ATP–pumping [48]) in the transition zone producing the ‘exited state’ 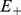. The system then returns (by spontaneous symmetry breaking), to the lower symmetry (cylindrical) state 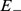, reducing the energy of the overall system stimulating axial expansion. Both transitions (up and down) complete one growth cycle with the predicted transition's rate 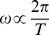, where 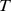 is the period. The whole process is repeated at the expense of pressure 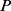 and ATP–energy needed for wall synthesis (exhibiting growth oscillations), and eventually expires or reaches critical instability at which point the cell bursts approximately at 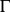, see SI: Movie S3 in [47].

In a mechanical anharmonic oscillator, the relationship between force and displacement is not linear but depends upon the amplitude of the displacement. In the case of the pollen tube the nonlinearity arises because of the visco-elastic flow/stretching of the wall material. The cell wall (acting as a spring component) is not capable of exerting a restoring force that is proportional to its displacement. As a result of this nonlinearity, the vibration frequency and amplitude can change, depending on the displacement of the system elements acting under pressure 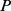. Appendix S1 in File S1 outlines an approximate derivation of the (dual) ‘frustration potential’, which is a sum of the attractive and repulsive forces, possibly responsible for experimentally observed growth rate oscillations in pollen tubes (we base our calculation on the local force equation of motion [49]). The oscillations given in Eq. (12) and visualized in Fig. 4, we describe thus: Pollen tube oscillations are trapped at the potential well about the equilibrium point 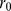 of the corresponding symmetric (harmonic) potential. Oscillation frequencies and amplitudes depend upon the turgor pressure values, see Eqs (6) and (7), as it is observed (e.g. Fig. 4 in [9]; [4]). Wall expansion is allowed by stress relaxation (molecular separation for 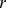 – values exceeding those of harmonic potential). Dissociation energy, at zero potential level, corresponds to system instability (burst at 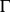). The low-lying (trapped) values deliver high frequencies and small amplitudes, while the higher-lying potential values – low frequencies and larger amplitudes of oscillations. Above the critical threshold (corresponding to ‘zero energy’ at the vertical scale) a bond breaking occurs and the pollen tube bursts at the transition zone, or delivers male gametes completing its function. The plot represents only one branch of the full frustration potential; the second branch is in ‘dual’ subspace, and the oscillations take place between both branches. For the negative values, the mechanism of symmetry breaking favorizes this ‘lower order’ (cylindrical) symmetry for cell extension.

The ‘frustration potential’ presented in Fig. 4 is a more convenient model for vibrational structure of wall constituing molecules than harmonic oscillator potential, because it explicitly includes the effects of bond breaking and accounts for anharmonicity of real bonds in the extending cell wall. It is also responsible for the inherent instability at the 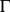 – interface of a growing tube (and – in consequnce – polymer building process), which can be experimentally supported by the fact that the pollen tubes almost always rupture at the transition zone where the radial part of the strain tensor is considerable (Movie S3 in [47]), or stress takes on the extreme value ([18], Fig. 5F – a yellow-orange coloured stripes). The form of the potential also contributes to the long debate among plant physiologists about the elastic/inelastic extension of plant cell wall in simple terms: Any departure from the parabola centered around 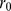 will lead to plastic extension, corresponding to elongation growth (see Fig. 4). In addition, at a given pressure 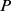 the infinite potential barrier at low distance 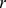 prevents the growing cell wall from shrinking.

In order to calculate the value of the angular resonance frequency 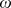 we momentarily accept the approximate (classical) relation: 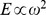. Assuming 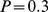 MPa, taking the approximate 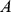 constant from the fit (see Fig. 5) we get 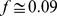 Hz, a value which belongs to the observed frequency spectrum in pollen tube growth functions (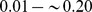 Hz, (see [14] for tobacco, Fig. 2; and [50] for lily). As an aside, we stress that the calculated from Eq. (7) resonance frequency satisfies a power law 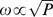 (see Appendix S1 for detailed derivation). The application of this important relation to the experimental data is presented in Fig. 5. From this discussion it is easy to see, that this relation (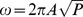, or equivalently 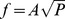, where 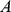 is a constant that is connected with the wall mechanical properties which can be determined from experiment, and [P] = MPa), if inverted, can serve (after calibration) to estimate turgor pressure 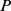 values from oscillation frequencies (or periods 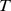) which are reatively easy to determine.

It is clear that the material properties of the cell wall in the apical region will not be homogeneous, and therefore a proper mechanical description of growth must involve a gradient in material properties from the apical to the distal region [18], [35]. It has been shown [35] that the calculated “expansion propensity” as a function of the distance from the apex measured in units of the tube radius 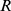 [35]) shrinks to an area near the apex. Closer examination reveals that the inflection point is located at about one pollen tube cylinder radius 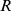, location targeted by our calculations. This means that the slope is the greatest at 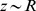 (in axial direction). This, and the fact that the ‘dilution’ sector is shown (Fig. 2 in [35]) exactly at the limit of the two considered axisymmetric zones, is consistent with the view that intense changes of the wall mechanical properties occur at the limits of the distal and apical parts. The ‘dilution’ effect is caused in our model by a rapid surface expansion due to displacement 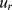 about the 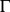 – interface. A corresponding radial strain may trigger exocytosis that results in delivery of new cell wall material which rejuvenates this area. This is also the conclusion of Geitmann and Dumais [42]. However, we should note that what we observe mainly reflects the changing symmetry at 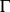 and the analytical consequences of curvature discontinuity (compare e.g. Eqs 3 and 4). These and other observations, [51], [42] indicate that exocytosis is likely to occur predominantly in the same annular region (cf. [52], Fig. 1) where wall expansion rates are greatest. The observation that tip growth is mainly confined to the annular region around the pole is in accord with calculations presented in this article (and also in [47], Movie S1 and S2, where tip growth ‘above’ the equator takes place).

Probing the mechanical properties of the “weld-zone” which joins the cylindrical and spherical parts may enable us to calculate the local rates of exocytosis, reversing the direction of causality implied by the conventional explanation for this phenomenon, where oscillating exocytosis rates are the cause of oscillating growth. The oscillation frequency can be estimated from Fig. 5 to be a value of approximately 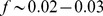 Hz, which is in accord with the main observed periodical mode [5] in the longitudinal power spectrum of pollen tube oscillatory motion, and presumably also with the rate of exocytosis and new cell wall assembly in *Nicotiana tabacum* pollen tubes [4]. This periodical growth activity, can in turn be related to the stress-relaxation rate at the 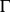 – interface (Fig. 3 in [53]). In addition, even small stress/strain fluctuations at this narrow cylindrical ring, could lead to macrosopic changes in the orientation of the ring and direction change of the elongating pollen tube as reported in Zonia and Munnik, Fig. 1F [29], and in Fig. 1 [54]. In general it seems that growth in pollen tubes is associated with spatially confined dynamic changes in cell wall mechanical properties ([55]; and File S1). For instance, the time course of an experiment, showing very slight changes in turgor pressure during cell growth was measured by pressure probe in growing *Lilium longiflorum* pollen tubes ([3], Fig. 1a) and re-analysed in ([7], Fig. 3b–c). Even though direct measurements failed to indicate large-scale turgor changes during growth, rapid small-scale pressure changes (jumps) are visible, which presumably were caused by changes in the orientation of the tilt angles of wall building cellulose microfibrils. (There is, however, no evidence for cellulose microfibrils to be involved in small changes in turgor. These small jumps in turgor may be simply imprecisions in the measurement method. This method requires readjusting the meniscus in a pressure probe needle for each data point and the measurement is inherently associated with significant noise). The direction of maximal expansion rate is usually regulated by the direction of net alignment among cellulose microfibrils, which overcomes the prevailing stress anisotropy [56]. As stated (ibid.), the measured periodicity for pressure oscillations ranges from 12 s to 25 s, which is the same as the routinely reported for oscillatory dynamics in lily pollen tubes [50], [55] and approximately agrees with the calculated frequency (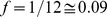 Hz) from our model. Closer examination of Figs. 3a–c in [7] reveals, in addition, a slight but steady diminishing of turgor (negative slope) which can be a consequence of cell volume expansion in every cycle. The latter observation may be associated with the passive role of the turgor pressure in pollen tube growth, at least at unchanging osmotic potentials. In conlusion, we agree with the view, that in pollen tube growth the energy supply is derived from turgor pressure, while the growth rate and direction is derived from the local wall properties [3].

It is clear that expansive growth in a plant cell relies on the interplay between the internal turgor and the forces in the cell wall opposing deformation. Which of these two parameters controls the dynamics of growth has been a matter of some controversy, particularly in the case of pollen tube growth [55]. The long-standing model considers that cyclic changes in cell wall properties initiate growth [57], [3]. A newer model based on accumulating data from recent work indicates that oscillations in hydrodynamic flow and intracellular pressure initiate growth [58], [7]. Both models agree that once growth initiated, osmotic pressure drives cell elongation. We have shown that the rapid growth phases during oscillatory growth in pollen tubes may be preceded by a strain – induced softening of the cell wall at the brink of the apical and distal parts (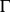). We also showed that cellular turgor pressure does not need to undergo changes during these repeated growth phases to display periodicity in growth. However, turgor pressure is still central in controlling the dynamics of pollen tube growth because of its ability to change wall stress and hence oscillation frequencies in the different osmotic environments [4].

There are many observations of collateral oscillations that could affect growth rates (such as oscillations in wall material deposition and/or extracellular ion fluxes). However, even from purely mechanical calculations, performed at the boundary between the wall cylinder shell and hemispherical shell at the apex, the following picture for the sequence of events for the elongating pollen tube emerges: Constant turgor pressure produces *different* strain rates in the apical and distal parts of the wall because they possess various mechanical properties and different symmetries. This phenomenon is amplified at the narrow interface between the cylindrical shank and the hemi-spherical tip. Consequently, a locally elevated stress is produced in the wall, in the order of tenths of a megapascal, causing serious instability at the brink of both sections, generated by symmetry frustration. The cell wall relaxation (loosening) process takes place so that to reduce tensile stress. This initiates a wall building process (which is an implicit assumption of the model) in meridional direction. However, as the tip continues to grow the frustration zone moves with it, and the whole process repeats itself. This results in time – periodicity in the growth dynamics recognized in the literature as pollen tube oscillations.

## Final Comment

This article offers a solution for the mechanism underlying observed pollen tube growth oscillations. It is based on the phenomenon of pressure – induced *symmetry frustration* of the cell wall in the apical region. The physical mechanism results in the appearance of quasi-discrete energy levels at (different) constant pressures in an analytically determined landscape of asymmetric topological potentials. Oscillatory growth is the inevitable outcome of the transitions between them. Moreover, a scaling relation between the turgor pressure 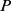 and the angular frequency of the oscillations 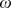 is derived, which is represented by a power 1/2 – law (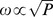). This prediction is successfully verified against real plant physiological experimental data. We hope this work will likely be appealing not only to plant physiologists but also to a physics audience who will appreciate the unique nature of instability as well as the analogy to pollen tubes.

## Supporting Information

File S1
**Appendix S1. Figure S1, Displacement ur[10−3 µm] due to the effective turgor pressure** 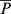 **(P = 0.3 MPa, pext = 0.05 MPa) acting on the cell wall as a function of the radial distance r from the pollen tube long axis. Figure S2, Tensile stress σrr (×10) due to the effective turgor pressure** 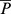 **acting on the cell wall at the position where (a) the cylinder (shank) joins (b) the hemisphere (apex) as a function of the radial distance r from the pollen tube axis. Figure S3, Tensile stress difference σrr(apex) − σrr(distal) at the apex and the distal part. Figure S4, Tensile stress difference** 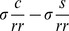 **(upper curves) and the opposite case** 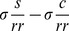 **(lower curves) calculated at the boundary zone between the approximately hemispherical apical and the cylindrical distal part of the growing pollen tube. Figure S5, Tensile stress difference** 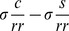 **(upper curves) and the opposite case** 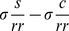 **(lower curves) calculated at the boundary zone between the semispherical apical and the cylindrical distal part of the growing pollen tube.**
(PDF)
