# Supplementary material for: Pressure–Induced Cell Wall Instability and Growth Oscillations in Pollen Tubes
Source: PLoS One. 2013 Nov 19;8(11):e75803. doi: 10.1371/journal.pone.0075803 (PMC3833986; doi:10.1371/journal.pone.0075803)
Supplement: File S1 — Appendix S1. Figure S1, Displacement ur[10−3 µm] due to the effective turgor pressure (P = 0.3 MPa, pext = 0.05 MPa) acting on the cell wall as a function of the radial distance r from the pollen tube long axis. Figure S2, Tensile stress σrr (×10) due to the effective turgor pressure acting on the cell wall at the position where (a) the cylinder (shank) joins (b) the hemisphere (apex) as a function of the radial distance r from the pollen tube axis. Figure S3, Tensile stress difference σrr(apex) − σrr(distal) at the apex and the distal part. Figure S4, Tensile stress difference (upper curves) and the opposite case (lower curves) calculated at the boundary zone between the approximately hemispherical apical and the cylindrical distal part of the growing pollen tube. Figure S5, Tensile stress difference (upper curves) and the opposite case (lower curves) calculated at the boundary zone between the semispherical apical and the cylindrical distal part of the growing pollen tube. (PDF) [file pone.0075803.s001.pdf]

Pressure – Induced Cell Wall Instability and Growth Oscillations in Pollen Tubes

Mariusz Pietruszka[1],
1 Mariusz Pietruszka, Faculty of Biology and Environment Protection, University of Silesia, Katowice, Poland
∗ **E-mail: mariusz.pietruszka@us.edu.pl**

Supporting Information

Quantitative calculations steming from Eqs (3) – (5) and originating from the gradient of mechanical properties at Γ are presented in SI Figs 1 – 5.

In SI Fig. 1 we observe (a) lowering of deformation u_r with radius r of apical hemispherical shell (b) almost constant u_r for a thin-walled cylinder. This fact produces a wall stress presented in SI Fig. 2 equal to about 100 MPa (for the model parameters), which seems big enough to cause cell wall instability at Γ – interface. Note, that the typical plant cell turgor pressures in the range of 0.3 to 1.0 MPa translate into between 10 and 100 MPa in the walls [39]. Hence, the calculated wall stress of the order of tenths of MPa is enough to cause local wall instability (radial strain), subsequent axial relaxation, wall building and unilateral cell expansion at the interface between the hemispherical apex and cylindrical shank. Unavoidable repetition of this process, owing to a constant effective pressure and a positive feedback mechanism necessary to drive oscillations (to overcome damping due to viscosity), may generate observable oscillations which continue until the wall building processes expire. Depending on the wall thickness, the calculated stress is equal to the distance between the curves (a) and (b) (inset). The tensile stress difference calculated at the apex and the distal part of the pollen tube cell wall (σ_{rr}(apex) – σ_{rr}(distal)) is shown in SI Fig. 3. Here it is parametrized by the turgor pressure P acting on the cell wall. The tensile stress in the wall clearly rises, as we increase the turgor pressure – this should, in turn, cause an increased growth oscillation frequency, as it is observed experimentally in the transition from hypertonic, through isotonic to hypotonic conditions [5].

The influence of turgor on the oscillation period, as predicted by the model described in [4], is also in agreement with our results presented in SI Fig. 4 and their consequences: the higher the pressure, the higher the oscillation frequency. Tensile stress difference at the transition zone, parametrized by the wall thickness, is presented in SI Fig. 5.

Stages of one oscillation (mechanical view):

1. Recovery through wall building at Γ – interface: visco-plastic process (elastic equations do not apply here, because of wall and mass production; the system is merely plastic and both subdomains are weakly coupled from the mechanical point of view).

2. Strain and deformation production on Γ (the equations apply).

3. Wall stress production on both sides of Γ and the resulting strain energy: visco-elastic process (equations apply).

4. Elastic strain energy relaxation in one cycle to produce elongation of one wave length (compare [34], Fig. 1C, Fig 6A): the phenomenology applies: $f = A\sqrt{P}$, finding confirmation in comparison with authors' performed experiment [14], Fig. 5 (article). The process repeats: next oscillation takes place (go to 1. to start another oscillation).

Pollen tube one oscillation: local deflection/wall stress/relaxation/recovery.

Appendix A

(i) The local force equation of motion in the mechanics of continuous bodies (due to Cauchy) reads [49]

$$\partial_j \sigma_{ij} + F_i = 0, \tag{1}$$

where i, j = 1, 2, 3, and Einstein's summation convention is used. By ignoring any possible shear stresses and assuming for the infinitesimally small $\Gamma -$ interface $\sigma_{\phi\phi} = 0$ and $\sigma_{zz} = 0$

$$\sigma_{ij} = \begin{pmatrix} \sigma_{rr} & 0 & 0 \\ 0 & \sigma_{\phi\phi} & 0 \\ 0 & 0 & \sigma_{zz} \end{pmatrix} \simeq \begin{pmatrix} \sigma_{rr} & 0 & 0 \\ 0 & 0 & 0 \\ 0 & 0 & 0 \end{pmatrix} \tag{2}$$

we receive $\sigma_{rr} = \sigma(r)$, which is the only matrix element that survives. From Eq. (8) we have

$$\int \partial_j \sigma_{ij} dx^i + \int F_i dx^i = 0 \tag{3}$$

so since $\int F_i dx^i = -U(x)$ is the potential energy, then $\int \partial_j \sigma_{ij} dx^i = U(x)$. Hence, in our case $\int \partial_r \sigma_{rr} dr = \sigma_{rr} = U(r)$, and one gets: $U(r) = \sigma_{rr} = \sigma(r)$.
(ii) By identyfying

$$U(r) = \sigma(r) \tag{4}$$

we may follow Eq. (6) to receive

$$\Delta\sigma^{\pm} = \pm \frac{\alpha}{r^3} \mp \frac{\beta}{r^2} + C, \tag{5}$$

where $\alpha = P\frac{(r_1 r_2)^3}{r_2^3 - r_1^3}$, $\beta = P\frac{(r_1 r_2)^2}{r_2^2 - r_1^2}$ and C is a constant. Next, in order to find r_0 we write $\Delta\sigma'(r_0) = 0$ to get

$$-\frac{3\alpha}{r^4} + \frac{2\beta}{r^3} = 0 \tag{6}$$

Hence

$$r_0 = \frac{3}{2}\frac{\alpha}{\beta}. \tag{7}$$

Since $U \equiv \Delta\sigma$ we can plot the potential $U(r)$, Fig. 4 in the main text.
(iii) Considering small oscillations around equilibrium r_0, we may introduce new coordinate ρ: $r = r_0 - \rho$ and by substituting it to Eq. (12) receive

$$U(x) = \frac{1}{r_0^3}\frac{\alpha}{(1-x)^3} - \frac{1}{r_0^2}\frac{\beta}{(1-x)^2}, \tag{8}$$

where $x = \rho/r_0$. By expanding both fractions for small x, we finally get the form for the harmonic potential:

$$U(x) = \frac{4}{9}\frac{\beta^3}{\alpha^2}x^2 - \frac{4}{27}\frac{\beta^3}{\alpha^2}. \tag{9}$$

Comparing the above equation with the classical oscillator potential ($m = 1$):

$$U(x) = \frac{1}{2}\omega^2 x^2 + U_0 \tag{10}$$

and using Eq. (12) we get $\omega^2 \propto P$.

Hence, the pollen tube oscillation frequency at the limit of small oscillations equals $\omega \propto \sqrt{P}$, where P is the turgor pressure (compare also with Fig. 5 in the main text, where the proportionality constant (A) is estimated from experiment).

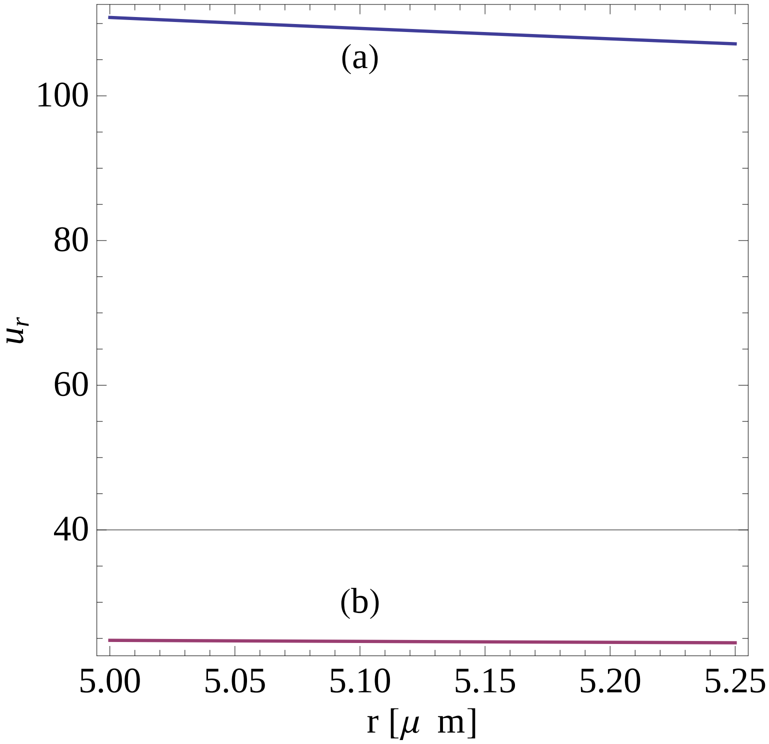

Figure 1. Displacement $u_r[10^{-3}\mu m]$ due to the effective turgor pressure $\tilde{P}$ ($P = 0.3MPa, p_{\text{ext}} = 0.05MPa$) acting on the cell wall as a function of the radial distance r from the pollen tube long axis. Different Young modulus ϵ and Poisson coefficient ν in both subsystems: (a) $u_r = u_r^s$ for a hemispherical apex: $r_1 = 5\ \mu m$, $r_2 = 5.25\ \mu m$, $\nu = 0.4$, $\epsilon = 0.2$ [GPa] (b) $u_r = u_r^c$ for a cylindrical distal part: $r_1 = 5\ \mu m, r_2 = 5.25\ \mu m$, $\nu = 0.2$, $\epsilon = 1$ [GPa].

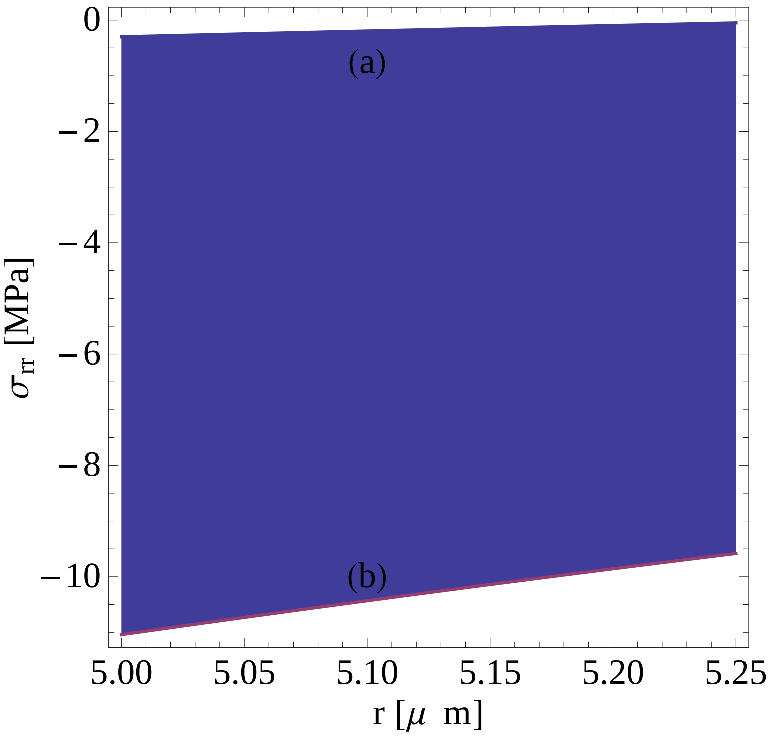

Figure 2. Tensile stress σ_{rr} (x 10) due to the effective turgor pressure $\tilde{P}$ acting on the cell wall at the position where (a) the cylinder (shank) joins (b) the hemisphere (apex) as a function of the radial distance r from the pollen tube axis. Radial stress discontinuity between the distal wall and apical wall is proportional to the distance between the curves (a) and (b). The calculated maximum wall stress reaches about 100 MPa for the simulation parameters. The strain energy leading to oscillations is proportional to the shaded area.

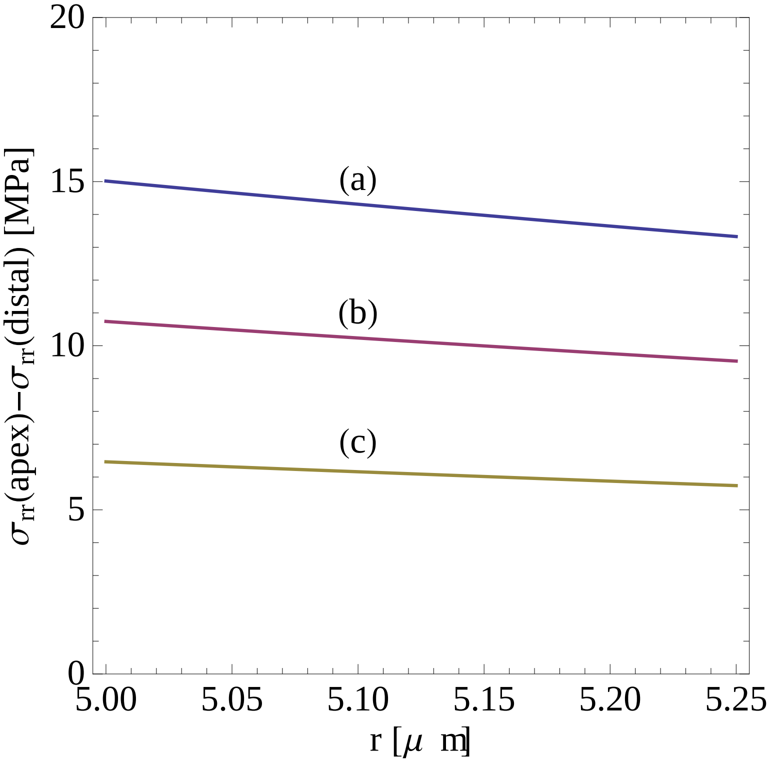

Figure 3. Tensile stress difference $\sigma_{rr}(\mathrm{apex}) - \sigma_{rr}(\mathrm{distal})$ at the apex and the distal part. Parametrisation by the changing turgor pressure P acting on the cell wall: (a) $P = 0.5$ MPa, (b) $P = 0.4$ MPa and (c) $P = 0.3$ MPa.

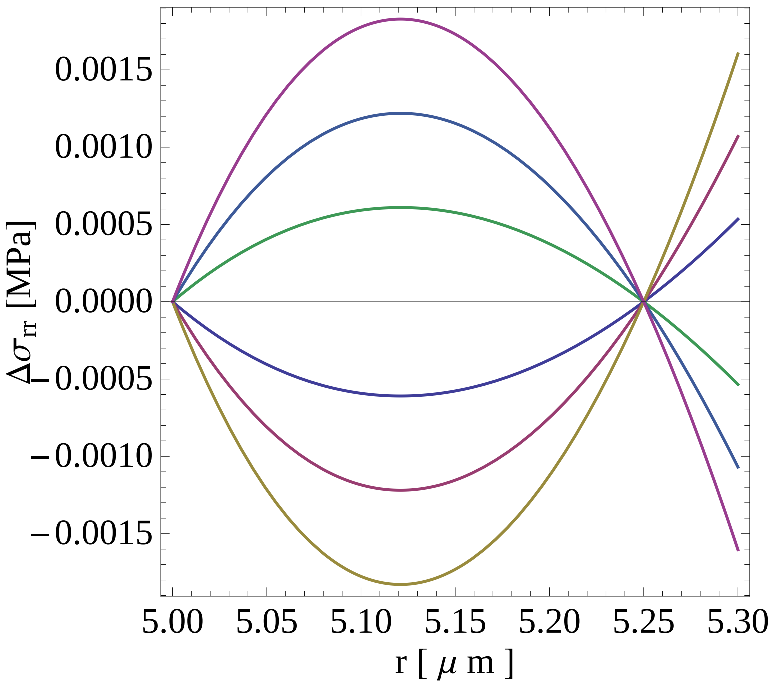

Figure 4. Tensile stress difference $\sigma^c_{rr} - \sigma^s_{rr}$ (upper curves) and the opposite case $\sigma^s_{rr} - \sigma^c_{rr}$ (lower curves) calculated at the boundary zone between the approximately hemispherical apical and the cylindrical distal part of the growing pollen tube. Parametrisation by the turgor pressure P acting on the cell wall (upper plots): $P = 0.1$ (green), $P = 0.2$ (blue) and $P = 0.3$ MPa (violet). Remaining parameters for the respective wall geometries: $r_1 = 5\ \mu m$, $r_2 = 5.25\ \mu m$.

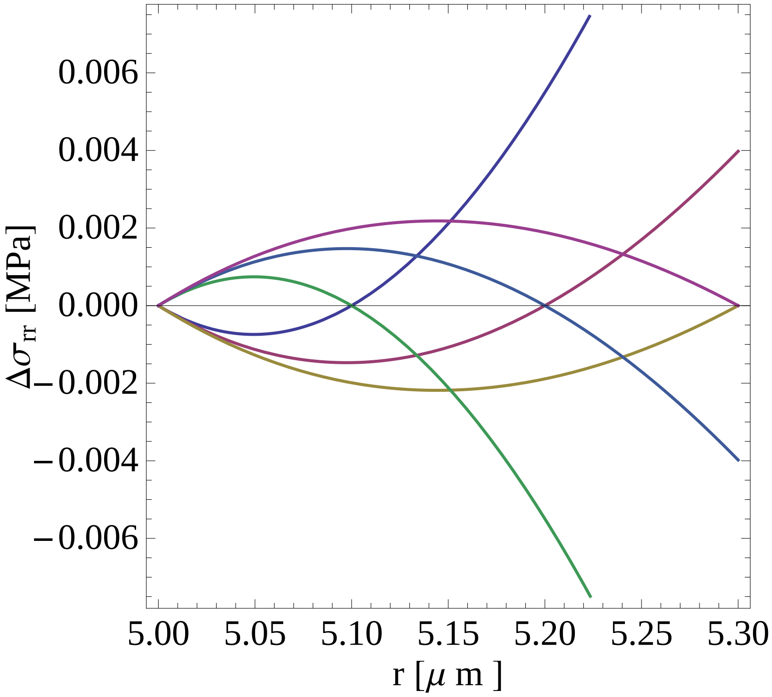

Figure 5. Tensile stress difference $\sigma^c_{rr} - \sigma^s_{rr}$ (upper curves) and the opposite $\sigma^s_{rr} - \sigma^c_{rr}$ (lower curves) calculated at the boundary zone between the semispherical apical and the cylindrical distal part of the growing pollen tube. Parametrisation by the wall thickness (upper plots): $r_2 = 5.1\ \mu m$ (green), $r_2 = 5.2 \mu m$ (blue) and $r_2 = 5.3\ \mu m$ (violet). The inner wall radius: $r_1 = 5\ \mu m$. Turgor pressure $P = 0.3$ MPa.
